# Whole transcriptome analysis reveals that immune infiltration- lncRNAs are related to cellular apoptosis in liver transplantation

**DOI:** 10.3389/fimmu.2023.1152742

**Published:** 2023-04-04

**Authors:** Shile Wu, Chao Cheng, Wenjun Zhu, Jinyu Yang, Bei-bei He, Song Li, Xinsheng Wang, Hao Guo, Dong Chen, Ya-min Guo

**Affiliations:** ^1^ Soochow University, Suzhou, Jiangsu, China; ^2^ General Surgery Department, Qinghai Provincial People’s Hospital, Xining, Qinghai, China; ^3^ Center for Genome Analysis, Wuhan Ruixing Biotechnology Co., Ltd, Wuhan, China

**Keywords:** liver transplantation, lncRNAs, immune cells, co-expression, apoptosis

## Abstract

**Introduction:**

In most instances, liver transplantation (LT) is the only available treatment for end‐stage liver diseases. However, LT could also induce serious liver diseases or injury, and the underlying mechanisms of LT-induced complications remain largely unknown, especially the mechanisms of the dysfunction of the immune system mediated by long noncoding RNAs (lncRNAs).

**Methods:**

In this study, we globally analyzed the proportion of immune cells by using the transcriptome sequencing data (RNA-seq) of needle-core liver biopsies from pre- and post-transplantation recipients. Dysregulated lncRNAs were found to be correlated with the altered fractions of immune cells. We finally explored the potential targets of dysregulated lncRNAs and analyzed their functions in LT.

**Results:**

We found that in the samples, some immune cells changed significantly after LT, including CD4 T cells, NK cells and mast cells. The proportion of macrophages in different polarization states also changed significantly, with M0 macrophages increasing and M2 macrophages decreasing. Through weighted gene co-expression network analysis (WGCNA), 7 gene expression modules related to LT were identified. These modules were related to changes in the proportion of different immune cells. The functions of these modules represent the response modes of different functional genes after LT. Among these modules, MEtan and MEyellow modules were primarily enriched in apoptosis and inflammatory pathways. Twelve immunity-related lncRNAs were identified for the first time, and the regulatory network co-changing with immune cells was also identified. The co-expressed genes of these lncRNAs were highly enriched in apoptosis-related pathways. Many apoptosis-related genes were found to be up-regulated after LT.

**Discussion:**

In summary, we speculated that the expression and regulation of these apoptotic genes may be related to the changes in the proportion of immune cells. Some of these lncRNAs and apoptosis-related genes have been reported to be related to cell proliferation and apoptosis. They are also potential biomarkers or therapeutic targets.

## Introduction

Liver diseases, which lead to end-organ failure, remain a growing cause of death. In most cases, the only treatment for liver failure is liver transplantation (LT) (1). However, there is an ineluctable risk of complications after LT, including ischemia-reperfusion injury (IRI) as the most common complication (2). Under ischemia-reperfusion stress, congenital adaptive immune crosstalk and cascade reaction of cell activation lead to liver inflammation-mediated injury (3). Many cellular and molecular mechanisms for controlling IRI in LT have been reported. These mechanisms involve a variety of cell types, such as sinusoidal endothelial cells, hepatocytes, neutrophils and platelets, and play a role through a network of interconnected molecular pathways, such as activating toll-like receptor (TLR) signals, changing the expression of microRNAs (miRNAs), producing reactive oxygen species (ROS), regulating autophagy and activating hypoxia inducible factors (2). The generation of ROS subsequent to reoxygenation causes tissue damage and triggers cell cascade reaction, leading to inflammation, cell death and organ failure (4). Increasing evidences indicate that Kupffer cells and T cells mediate the activation of neutrophil inflammatory responses. Activated neutrophils were infiltrated into the damaged liver, and the expression of adhesion molecules on endothelial cells increased (5). The heme oxygenase (HO) system, one of the most critical cell protection mechanisms activated during cell stress, plays an anti-oxidant and anti-inflammatory role, regulates cell cycle and maintains microcirculation (6). The activation of TLR on Kupffer cells may provide a trigger signal for proinflammatory response in ischemia/reperfusion (I/R) injury. Dissecting TLR downstream signaling pathways plays a fundamental role in exploring new therapeutic strategies based on the concept that liver I/R injury represents a case for host “innate” immunity (7).

Long noncoding RNAs (LncRNAs) are considered to be important regulatory transcripts in various tissues and organs, and may play an important role in the regulation of many biological processes (8). LncRNAs also play an important role in liver IRI (9). Under hypoxia/reoxygenation (H/R) conditions, the expression of lncRNA-*MALAT1* in human hepatocytes increased. MALAT1 may aggravate liver I/R injury by regulating apoptosis and inflammation triggered by HMGB1-TLR4 (10). In addition, lncRNA-*TUG1* has a protective effect on cold-induced liver injury in mice by inhibiting cell apoptosis, suggesting that TUG1 is a potential target to prevent cold-induced liver injury in LT (11). It was reported that after IRI, the expression of lncRNA-*AK054386* was up-regulated in liver IRI models, and the silencing of liver *AK054386* attenuated the liver IRI (12). At present, some studies identified dysregulated lncRNAs induced by IRI. A study suggests that plasma LncRNA expression disorders may act as new biomarkers for evaluating ischemic liver injury (13). LncRNAs are multifunctional molecules, which can interact with RNAs, DNAs or proteins to promote or inhibit the expression of protein-coding genes. The activation of immune cells is related to the dynamic changes of gene expression. The products of gene expression fight against infectious microorganisms, initiate repair, and relieve the inflammatory reaction in cells and tissues. Recent evidence confirms that lncRNAs play an important role in guiding the development of various immune cells and in controlling the dynamic transcription of immune cell activation markers (14). In addition, lncRNAs may be key regulators in innate and adaptive immune response systems (15). Therefore, an in-depth understanding of the molecular mechanism of lncRNAs in immune response may contribute to the development of potential therapeutic targets for the treatment of various diseases (16). However, how lncRNAs affect IRI by regulating the immune microenvironment during LT remains unclear.

In this study, we speculate that the expression of lncRNAs and genes controlling the survival of immune cells will be aberrant before and after LT. At the same time, as an important regulator of immune cells, the aberrant expression of lncRNAs may affect and reshape the composition of immune microenvironment in the pre and post LT. We downloaded the RNA-seq data of 40 pre- and 40 post-transplant liver tissues from GSE151648 (17), and analyzed the composition of immune cells. We then systematically investigated the lncRNA profiles and explored the relationship between immune cell composition and dysregulated lncRNAs. A large number of apoptosis-related genes were found to be up-regulated after LT. Some of these lncRNAs and apoptosis-related target genes have been reported to be related to cell proliferation and apoptosis. They are also potential biomarkers or therapeutic targets.

## Materials and methods

### Retrieving and processing public data

Public sequence data files from GSE151648 dataset (17) were downloaded from the Sequence Read Archive (SRA). SRA Run files were converted to fastq format with NCBI SRA Tool fastq-dump. Low-quality bases were removed from the raw reads using the FASTX-Toolkit (v.0.0.13; http://hannonlab.cshl.edu/fastx_toolkit/). Then the clean reads were evaluated using FastQC (http://www.bioinformatics.babraham.ac.uk/projects/fastqc).

### Reads alignment and analysis of differentially expressed genes

Clean reads were aligned to the human GRCH38 genome by HISAT2 (18), with no more than 4 mismatches. Uniquely mapped reads were eventually used to calculate reads number and reads per kilobase of exon per million fragments mapped (FPKM) for each gene. The expression levels of genes were evaluated using FPKM. The software DESeq2 was used to predict DEGs, (19) with criteria set as a fold change (FC) ≥ 2 or ≤ 0.5 and false discovery rate (FDR) ≤ 0.05.

### Cell-type quantification

The CIBERSORT algorithm (v1.03) (20), together with the FPKM value of each expressed gene, was used to estimate immune cell fractions. The parameters were set to default values. A total of 22 immune cell phenotypes were analyzed, comprising 6 T cell types [CD8 T cells, naïve CD4 T cells, memory CD4 resting T cells, memory CD4 activated T cells, T follicular helper cells, and regulatory T cells (Tregs)]; naïve and memory B cells; plasma cells; resting and activated NK cells; monocytes; macrophages M0, M1, and M2; resting and activated dendritic cells; resting and activated mast cells; eosinophils; and neutrophils.

### LncRNA prediction and direction identification

To analyze lncRNA expression pattern, we used a pipeline to identify lncRNAs as previously reported (21). Briefly, we used StringTie software (22) to assemble the data of each group and predict the transcripts, screened the expression of the predicted transcripts of each group, eliminated the transcripts with FPKM< 1, and then used StringTie to combine them into one transcript (GTF file). Four software was used to predict the coding potential of lncRNAs: CPC2 (23), LGC (24), CNCI (25), and CPAT (26). We then counted the noncoding transcripts identified by the above four analysis software. After the above steps, we successively removed the transcripts that overlap with the known coding genes, are less than 200bp in length, have potential coding ability, and are less than 1000bp away from the nearest gene from the assembly results, obtained the prediction results of new lncRNA, and used the intersection of the four software for subsequent analysis and processing.

### WGCNA and co-expression analysis

Using default parameters, weighted gene co-expression network analysis (WGCNA) was conducted (27) to fully understand the expression pattern of all expressed genes and to cluster genes having similar expression patterns. All expressed genes (25% of samples with FPKM>=0.5 and at least one sample with FPKM>=1) in 80 samples were used as input data. The eigengene served as the representative of the gene expression profiles in the cluster. Module–trait associations were also investigated using WGCNA. To explore the regulatory mode between lncRNAs and their target genes, we calculated the Pearson’s correlation coefficients (PCCs) and divided their relation into three categories based on the PCC values: positively correlated, negatively correlated and non-correlated.

### Functional enrichment analysis

To sort out functional categories of DEGs, Gene Ontology (GO) terms and Kyoto Encyclopedia of Genes and Genomes (KEGG) pathways were identified using the KOBAS 2.0 server (28). Hypergeometric test and Benjamini-Hochberg FDR controlling procedure were used to define the enrichment of each term.

### Other statistical analysis

R package factoextra (https://​cloud.r-project.org/​package=factoextra) was used to perform Principal component analysis (PCA) to show the cluster of samples with the first two components. The pheatmap package (https://cran.r-project.org/web/packages/pheatmap/index.html) in R was used to perform cluster analysis based on Euclidean distance. Student’s *t*-test was used to compare two groups.

## Results

### Diversity of immune microenvironment characteristics before and after LT

**Figure 1:**
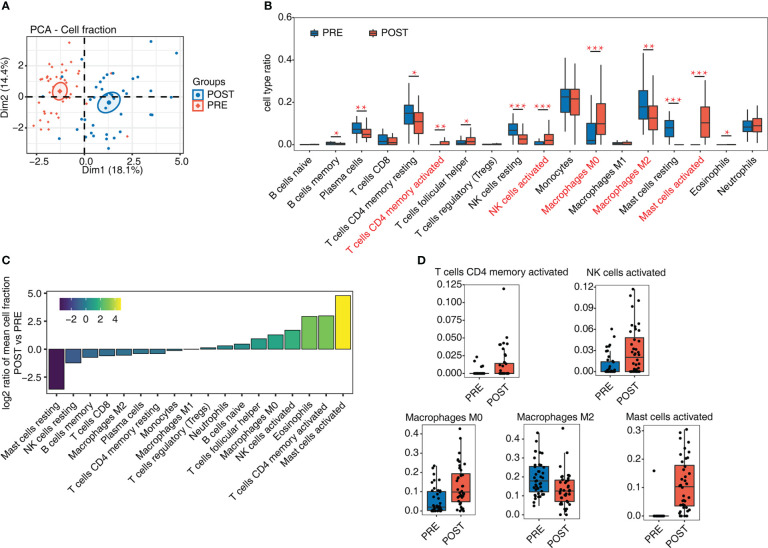
Diversity of immune microenvironment characteristics before and after LT. **(A)** PCA based on the fractions of different cells before and after LT. The ellipse for each group is the confidence ellipse. **(B)** Box plot shows the fraction of each cell type in each group. Student’s *t*-test is used to calculate the significant differences of cell fractions between PRE and POST samples. *P-value < 0.05, **P-value < 0.01, ***P-value < 0.001. **(C)** Bar plot shows the relative frequency ratio between POST *vs.* PRE for each cell type that is ranked based on decreasing values. **(D)** Box plot shows the fraction distribution of 5 immune cell types.

The success of LT and the following immune tolerance could be controlled by various types of immune cells. To decipher how immune cells were changed after LT, we analyzed whole transcriptome sequencing data (RNA-seq) from GSE151648 (17). The data comprise 40 pre-transplant (PRE) and 40 post-transplant (POST) living liver samples. After obtaining the expression levels of all detected genes, we used CIBERSORT software (20) to analyze the fractions of 22 dominant hematopoietic cell types. The PCA result of these 22 cell fractions demonstrated that PRE and POST samples can be separated by the first component (**Figure 1A**), indicating that the composition of immune cells has been greatly changed after LT. Then 4 cell types were removed due to their low detection rate (< 10% of samples). Based on the percentage of these 18 cell types, several cell types were found to have high levels, including monocytes (~21.06%), macrophages (~26.48%), and T cells (~17.50%). There were also individual variations across the samples (**Figure S1A**). We then drew a statistical comparison of all cell types between PRE and POST samples, and found 12 cell types showed significant differences (**Figure 1B**), suggesting the proportions of immune cells in POST samples were dysregulated. The increase of the activated cell types was higher, including CD4 memory T cells, NK cells, and mast cells, while the resting cell types decreased in POST samples (**Figures 1B, C**). The proportion of macrophages in different polarization states also changed significantly, with M0 macrophages increasing and M2 macrophages decreasing (**Figures 1B, C**). These results demonstrated that the cell fraction and the cellular states of immune cells were dysregulated after LT. The detailed fractions of several fraction dysregulated cell types are presented to show the obvious differences between PRE and POST samples (**Figures 1D**, **S1B**).

### Genome-wide profiling of the immune-associated lncRNA expression before and after LT

**Figure 2:**
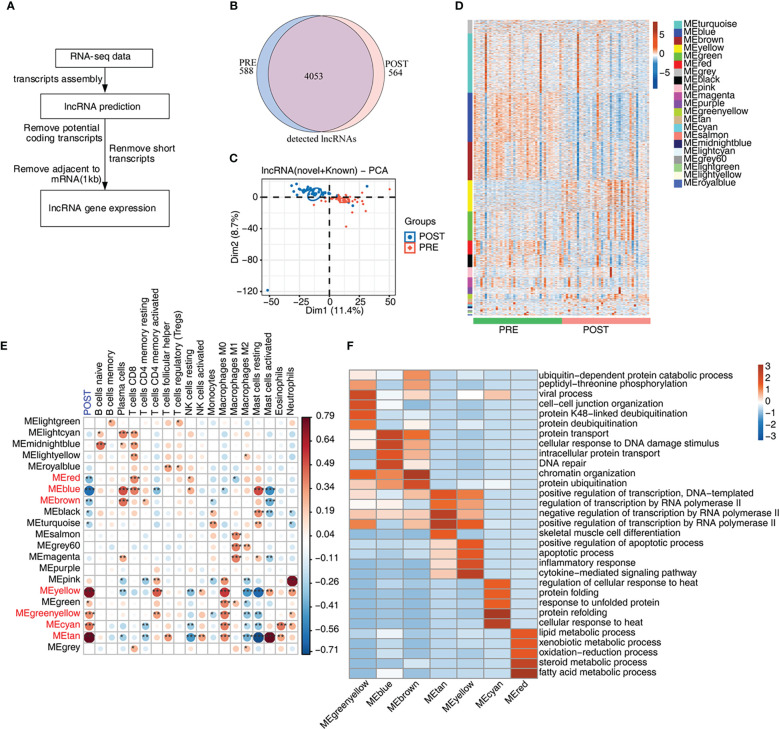
Genome-wide profiling of the immune-associated lncRNA expression before and after LT. **(A)** **Figure 2A** presents bioinformatics analysis pipeline for the identification of lncRNAs. **(B)** Venn diagram shows the number of detected lncRNAs. The known and newly predicted lncRNAs are overlapped, and more than 1 samples with FPKM < = 0.2 were supposed to be detected. **(C)** PCA based on FPKM values of all detected genes. The ellipse for each group is the confidence ellipse. **(D)** WGCNA analysis of all expressed mRNAs and lncRNAs is performed. Heat map displays lncRNA and mRNA expression levels according to WGCNA modules. **(E)** WGCNA module-trait associations are plotted using ‘plotEigengeneNetworks’ function with all factors (LT status and cell fractions) on the x-axis acting as covariates. Modules in red font were LT-associated modules. The colors indicate Pearson’s correlation values and p values are displayed with stars. *p-value < 0.05; **p-value < 0.01; ***p-value < 0.001. **(F)** The heat map shows that the GO items (BPs) of 7 module traits with WGCNA *p*-value < 0.01.

Recent studies have shown that lncRNAs modulate the homeostasis and functions of immune cells (14, 29), suggesting expression of lncRNAs may be dysregulated and may participate in the immune response in LT. We then explored the expression profiles and potential functions of lncRNAs in PRE and POST samples (**Figure 2A**). Initially, we predicted novel lncRNAs by constructing novel transcripts and calculating their coding potential. After removing suspicious codes or transcripts that are too short, we obtained all lncRNAs and their expression levels. We found that the co-detected lncRNAs were dominant both in PRE and POST samples (**Figure 2B**), and that this feature was present both in known lncRNAs and novel lncRNAs (**Figures S2A, B**). We compared other features of lncRNAs with those of mRNAs, and found that lncRNAs had fewer exons and shorter transcripts than that of mRNAs (**Figures S2C, D**), indicating the two groups had distinct transcription features. Although these lncRNAs were detected both in PRE and POST samples, PCA result showed PRE and POST samples had separable expression patterns in the first component (**Figure 2C**), suggesting that lncRNA expression pattern was dysregulated in POST samples.

To further explore the expression feature of lncRNAs, we performed WGCNA of all detected mRNAs and lncRNAs, and obtained 21 expression modules with specific expression patterns (**Figure 2D**). Several modules, such as yellow and green modules, showed higher expression levels in POST samples (**Figure 2D**). According to the number of lncRNAs in each module, lncRNAs in these 21 modules were gradually decreased (**Figure S2E**). To investigate the relationship between expression modules and dysregulated immune cell types, we performed correlation analysis. Seven modules were found to be correlated with the features of POST samples and were considered to be LT-associated modules (**Figure 2E**, red font), comprising 3 negatively correlated modules (blue color) and 4 positively correlated modules (red color). We also constructed the correlation network between the expression modules and immune cell types, and found that most modules were correlated with at least one immune cell type. Meanwhile, several immune cells, including CD8^+^ T cells, M0 and M2 macrophages, and mast cells were correlated with more expression modules than other immune cells, suggesting these correlated expression modules have potential influences on immune cells (**Figure 2E**). Finally, we performed functional enrichment analysis of the 7 LT-associated modules, and found they were enriched in distinct functional pathways by performing GO (**Figure 2F**) and KEGG (**Figure S2F**) enrichment analysis. Genes in MEcyan, MEgreenyellow, MEtan and MEyellow modules were up-regulated after LT. MEtan and MEyellow modules were primarily enriched in apoptosis and inflammation pathways; MEcyan module was primarily enriched in heat response and protein folding pathways; MEgreenyellow module was primarily enriched in protein ubiquitination- related pathways (**Figure 2F**). Genes in MEblue and MEbrown modules were down-regulated, and they were chiefly enriched in DNA damage response-related pathways; genes in the MEred module were also down-regulated, and were primarily enriched in lipid/steroid/fatty acid metabolism and other related pathways (**Figure 2F**).

### Construction of co-expression network between immune-related lncRNAs and DEGs involved in apoptosis

**Figure 3:**
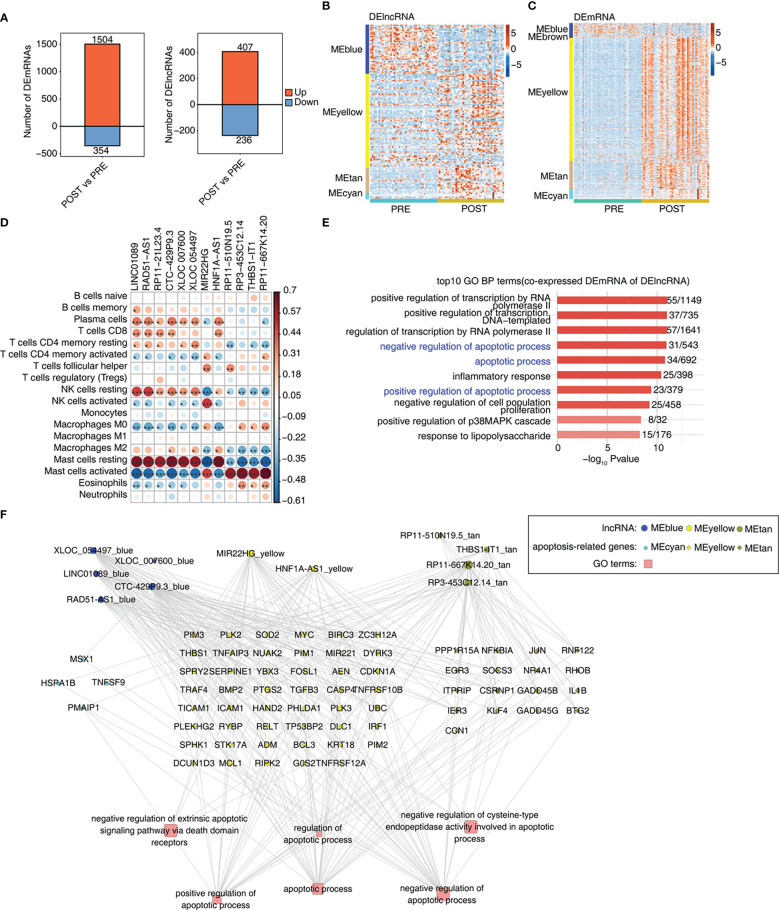
Construction of co-expression network between immune-related lncRNAs and DEGs involved in apoptosis. **(A)** Bar plot shows the number of up- and down-regulated lncRNAs (DElncRNAs) and annotated genes (DEG). DESeq2 is used for differential expression analysis with the criteria set to a *p*-value ≤ 0.01 and FC ≥ 2 or ≤ 0.5. **(B)** Heat map shows the expression levels of DElncRNAs in WGCNA module trait with a *p*-value ≤ 0.01. **(C)** Heat map shows the expression levels of DEmRNAs in WGCNA module trait with a *p*-value ≤ 0.01. **(D)** Co-expression analysis of DE lncRNAs with at least one immune cell with a *p*-value and Pearson’s correlation value ≥ 0.6 or ≤ -0.6. **(E)** Co-expression analysis of the DEmRNAs from modules in **Figure 3C** and of DElncRNAs in **Figure 3D**. Cutoffs of p-value ≤ 0.01 and Pearson coefficient ≥ 0.6 or ≤ -0.6 are applied to identify the co-expression pairs. Bar plot exhibits the top GO BP results of co-expressed DEmRNA genes. **(F)** Network diagram displays the apoptotic pathways of DEmRNAs regulated by DElncRNAs. *p-value < 0.05; **p-value < 0.01; ***p-value < 0.001.

We further explored the differentially expressed mRNAs (DEmRNAs) and lncRNAs (DElncRNAs) between POST and PRE samples. Using the criteria of FC > 2 and FDR < 0.05, we identified in POST samples more up-regulated mRNAs and lncRNAs than down-regulated ones. And more DEmRNAs were identified than DElncRNAs (**Figures 3A**, **S3A**). We then extracted DElncRNAs and DEmRNAs from the 7 modules associated with LT. DElncRNAs and DEmRNAs from MEblue module were down-regulated in POST sample., Most of the DElncRNAs and DEmRNAs from MEyellow, MEtan, and MEcyan modules were up-regulated in POST samples (**Figures 3B, C**). To construct the potential regulatory network, we analyzed the correlation between LT-associated DElncRNAs and immune cell types. With the criteria set to a *p*-value < 0.01 and Pearson’s correlation coefficient > 0.6, we identified 12 DElncRNAs that showed a significant correlation with at least one immune cell (**Figure 3D**). The proportion dysregulated immune cell types, especially for mast cells, were also correlated with the DElncRNAs (**Figure 3D**). We found 7 DElncRNAs were correlated to resting mast cells and 5 DElncRNAs were correlated to activated mast cells (**Figure 3D**), suggesting DElncRNAs are potential regulators of immune cell composition and function. To identify the potential functions of these 12 DElncRNAs, we performed functional enrichment analysis of their co-expressed mRNAs. In addition to the transcription regulatory pathways, the apoptosis-associated pathways were also enriched (**Figure 3E**). Moreover, inflammatory-associated pathways were in the top GO BPs and KEGG pathways (**Figures 3E**, **S3B**). We finally constructed the lncRNA-mRNA-pathway regulatory network for the enriched apoptosis pathways and the contained mRNA genes. DElncRNAs were from MEblue, MEyellow, and MEtan modules; DEmRNAs were from MEcyan, MEyellow, and MEtan modules (**Figure 3F**). The enriched apoptosis-related pathways of DElncRNAs suggest that these lncRNAs may affect the survival of immune cells by regulating the expression of apoptosis-related genes.

### Genes involved in apoptosis pathway were largely up-regulated by 12 immune-associated DElncRNAs after LT

**Figure 4:**
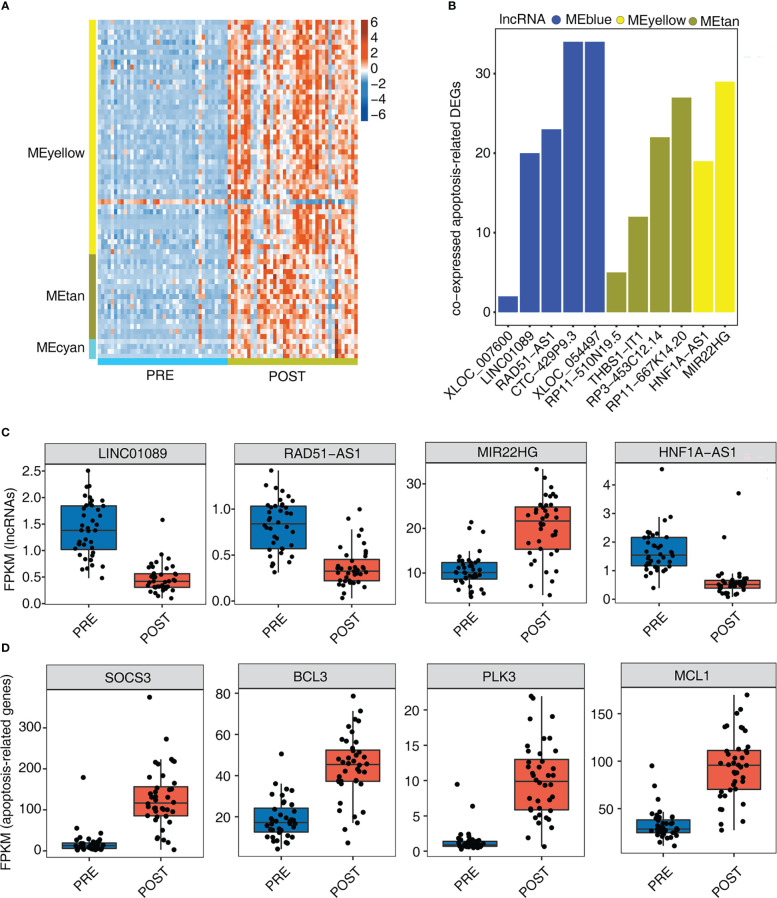
Genes involved in apoptosis pathway are largely up-regulated by 12 immune- associated DElncRNAs after LT. **(A)** The heat map shows the gene expression profiles of apoptosis-related DEGs in **Figure 3F**. **(B)** The bar graph shows the number of DEGs that were co-expressed by DElncRNAs and involved in apoptotic pathways with Pearson’s correlation value > = 0.6 or < = - 0.6 and *p*-value<=0.01. **(C)** Box plot shows the expression profiles of 4 immune-related lncRNAs. **(D)** Box plot shows the expression profiles of 4 apoptosis-related genes co-expressed by DElncRNAs.

Necroptotic cell death and macrophage apoptosis have important influence in LT-induced IRI (30, 31). In this part, we therefore focused on the identified apoptosis pathways and their associated genes (**Figure 3F**). By extracting apoptosis-associated genes from MEyellow, MEtan, and MEcyan modules, we found that these genes were expressed at higher levels in POST samples than in PRE samples (**Figure 4A**), indicating that these up-regulated apoptosis genes may be involved in the adaptive process after LT. To further investigate whether these apoptosis genes were regulated by lncRNAs, we further identified the number of correlated apoptosis genes of each lncRNA, and found that 11 lncRNAs showed a correlation with apoptosis-related genes; these 11 lncRNAs originated from three modules: MEblue, MEyellow, and MEtan (**Figure 4B**). Among these lncRNAs, *CTC-429P9.3*, *XLOC_054497*, *RP11-667K14.20*, and *MIR22HG* were correlated with more apoptosis genes than other lncRNAs (**Figure 4B**). **Figures 4C**, **S4A** present the detailed expression level changes of these 11 lncRNAs and the changed patterns between POST and PRE samples. Meanwhile, we also presented the apoptosis-associated genes that were potentially regulated by these 11 lncRNAs. Four of these apoptosis-associated genes, including SOCS3, BCL3, PLK3, and MCL1, showed the elevated gene expression levels of the selected apoptosis genes in POST and PRE samples (**Figure 4D**).

## Discussion

Several advances have been made in the field of LT over the last two decades. However, extensive application is restricted due to essential unsolved problems and challenges (32). The molecular mechanisms underlying LT-induced risks are complex, including the dysfunction of immune cells, alteration in gene expression, and unbalanced cellular metabolism (2). Recent studies have summarized the important roles of lncRNAs and miRNAs in the pathological progression of LT, a common complication of liver surgeries (33, 9). Based on these questions, in this study, we explored the expression and proportion dysregulated lncRNAs and immune cell types after LT, respectively, and constructed a network of these lncRNAs and immune cells. The results suggested that DElncRNAs may have potential roles in modulating the proportions of immune cells and regulating the expression of apoptosis-associated genes, which could be involved in the progression of diseases induced by LT.

The composition and status of immune cells can monitor post-LT infection and patient conditions, suggesting the potential application in diagnosis and therapy for LT patients (34). Regulatory T cells (Tregs) have been used as immunotherapy for LT patients and can reduce anti-donor T cell responses (35). Thus, we investigated the immune cell alteration between PRE and POST samples. Compared with in PRE samples, many types of immune cells were found to be activated in POST samples, indicating that immune cells were involved in post-LT immune responses or tolerance. Mast cells were the most dysregulated cells and were completely activated and lost stabilization after LT. A previous study demonstrated that mast cell stabilization could downregulate pro-inflammatory cytokine levels and attenuate acute lung injury caused by LT, suggesting that activated mast cells may contribute to post-LT inflammation (36). Resting and activated natural killer cells also exhibited converse changes after LT, implying they regulate post-LT immune tolerance and influence LT outcomes (37). In this study, we also detected the alteration of the two types of macrophages (**Figure 2D**). Macrophage polarization and apoptosis have been considered to be important topics concerning post-LT IRI (31). The anti-inflammatory M2 macrophages were repressed while pro-inflammatory macrophages increased after LT, suggesting the circulating macrophages are involved in post-LT immune responses. Macrophage activation and IRI were inhibited in mouse orthotopic liver transplantation (MOLT) by targeting TIM-1 on CD4 T cells, indicating that immune cells are appropriate candidates for LT-induced IRI therapy (38). In this study, we demonstrated the essential roles of the activated immune cells in immune or inflammatory responses to LT. These immune cells may act as therapeutic targets for post-LT immune tolerance. Further studies, especially the application of single cell technologies and external cohort of validation, are necessary to explore the biological functions of certain immune cells.

We then investigated the expression dysregulated lncRNAs in POST samples. Although most lncRNAs were identified in both PRE and POST samples, their expression patterns were dramatically changed after LT. We identified several lncRNA and mRNA modules correlated with the POST status and cell types of LT patients, indicating dysregulated lncRNAs play important roles in immune responses induced by LT. A recent study has summarized the significant changes of lncRNAs and miRNAs and their potential for prognostic markers in IRI caused by LT (9). A previous study demonstrated that lncRNA MIR155HG can regulate macrophage polarization in chronic obstructive pulmonary disease (COPD) (39). Integrating bioinformatics analysis with multiple datasets, Zhou et al. identified 6 immune-related lncRNAs that were associated with the therapeutic outcomes and immune microenvironment of hepatocellular carcinoma (HCC) patients (40). In this study, by analyzing the enriched functions of the 7 WGCNA modules, we found they were enriched in several pathways, including inflammatory response, apoptotic process, and transcriptional regulation, suggesting the dysregulated lncRNAs may participate in the essential biological pathways associated with immune cell dysregulation. However, we also realize that these results are only based on the bioinformatics analysis, and needed to be validated using other biological technologies in future studies.

Then we identified the DElncRNAs and explored their potential functions. We finally identified 12 lncRNAs showing a correlation with dysregulated immune cell types. Almost all the co-expressed mRNAs of these DElncRNAs were up-regulated in POST samples, indicating DElncRNAs may upregulate DEmRNAs. By analyzing the functions of these DEmRNAs co-expressing with DElncRNAs, we found they were significantly enriched in apoptosis-associated pathways. Dysregulation of apoptosis plays a central role in many disease processes pertinent to LT, including immune modulation, preservation injury, and viral diseases (41). In addition, the apoptosis of sinusoidal endothelial cells plays critical roles in preservation injury in rat liver transplantation (42). Among the dysregulated lncRNAs, we found several lncRNAs have been reported with apoptosis. LncRNA *RAD51-AS1* has been reported to be upregulated in epithelial ovarian cancer (EOC); silencing *RAD51-AS1* inhibited EOC cell proliferation, delayed cell cycle progression and promoted apoptosis *in vitro* and *in vivo* (43). MIR22HG expression is elevated in many human cancers and is involved in many signaling pathways by acting as a competitive endogenous RNA, such as cell proliferation and apoptosis pathways (44). LncRNA *LINC01089* inhibits cell proliferation and promotes the apoptosis of lung adenocarcinoma (LUAD) cells *via* acting as a ceRNA (45). Together, these results indicate that the dysregulated lncRNAs may be involved in post-LT immune tolerance or injury progression by modulating the immune cell status and apoptosis levels.

In conclusion, our study highlighted the expression dysregulated lncRNAs after LT and explored their association with proportion dysregulated immune cells related to post-LT immune tolerance and IRI. Several lncRNAs may be involved in this progression by modulating the expression of apoptosis-related genes; and they can serve as novel therapeutic or diagnostic targets for immune resistance or post-LT IRI treatment. These bioinformatics prediction results inspire us that further studies are necessary to explore the molecular mechanisms of these dysregulated lncRNAs.

## Data availability statement

The original contributions presented in the study are included in the article/**Supplementary Material**. Further inquiries can be directed to the corresponding authors.

## Author contributions

S-LW, CC: Article design, writing data collection and analysis. W-JZ, J-YY, SL, B- BH, X-SW: Article writing, data collection, literature retrieval. HG: Data analysis. Y-MG, DC: Data review and article verification. All authors contributed to the article and approved the submitted version.
