# Supplementary material for: Whole transcriptome analysis reveals that immune infiltration- lncRNAs are related to cellular apoptosis in liver transplantation
Source: Front Immunol. 2023 Apr 4;14:1152742. doi: 10.3389/fimmu.2023.1152742 (PMC10110847; doi:10.3389/fimmu.2023.1152742)
Supplement: Supplementary file 1 [file DataSheet_1.docx]

**Supplementary Figures**


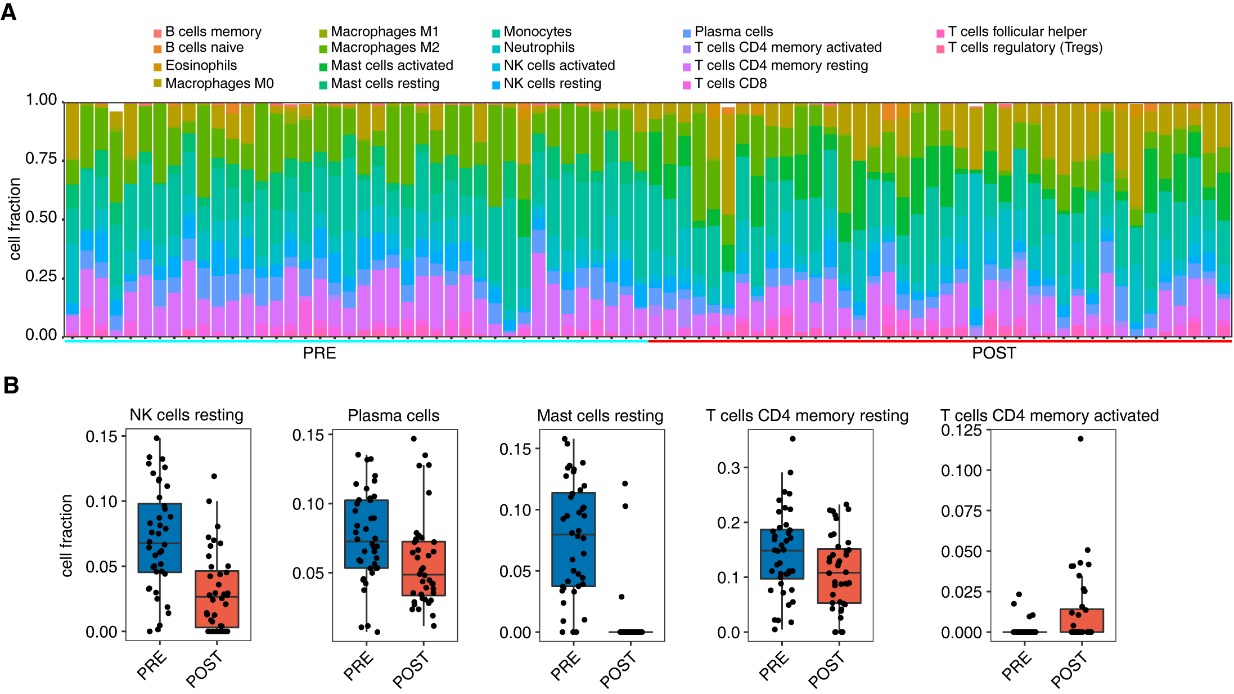


**Figure S1. Diversity of immune microenvironment characteristics before and after liver transplantation.**

1. Fractions of different cells estimated by CIBERSORT in each sample.

B. Boxplot showing cell fraction of five cell types.

**
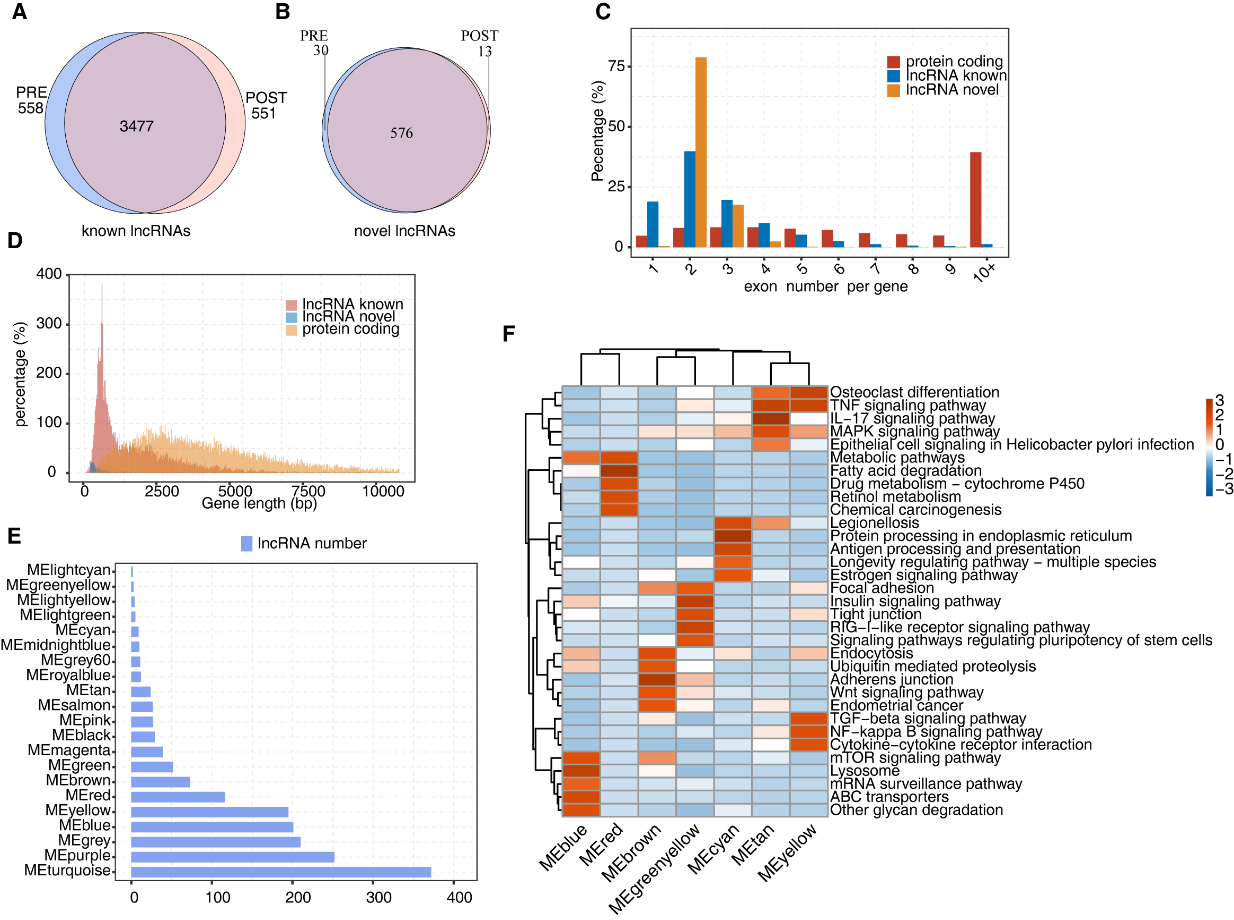
**

**Figure S2. Genome-wide profiling of the immune-associated lncRNA expression before and after liver transplantation.**

1. B. Venn diagram of Known (left)and novel (right) lncRNA genes in PRE and POST samples, Shock P. At least two samples with FPKM>=0.2 is considered to be detected in the group.

C. Distribution of exon length of known lncRNA, novel lncRNA, and protein coding RNA.

D. Density of the length distribution of known lncRNA, novel lncRNA, and protein coding

RNA. The length density distribution was generated by density function in R.

E. The bar graph showing the number of lncRNA in WGCNA module-trait.

F. The heatmap shows that the KEGG enrichment pathway of 7 module traits with WGCNA p value ≤ 0.01.


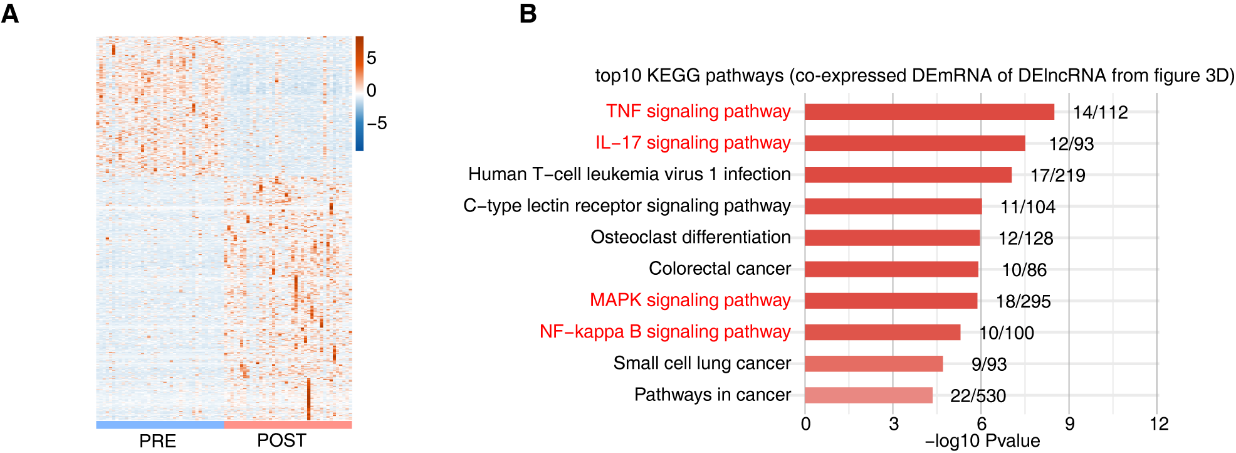


**Figure S3. Construction of co-expression network between immune-related lncRNA and DEGs involved in apoptosis.**

1. Heatmap showing the expression level of DElncRNA.
2. Co-expression analysis was performed on the DEmRNA from modules in figure 3C of DElncRNA in figure 3D. Cutoffs of p value ≤ 0.01 and Pearson coefficient ≥ 0.6 or ≤ -0.6 were applied to identify the co-expression pairs. Bar plot exhibites the KEGG enrichment pathway results of co-expressed DEmRNA genes.


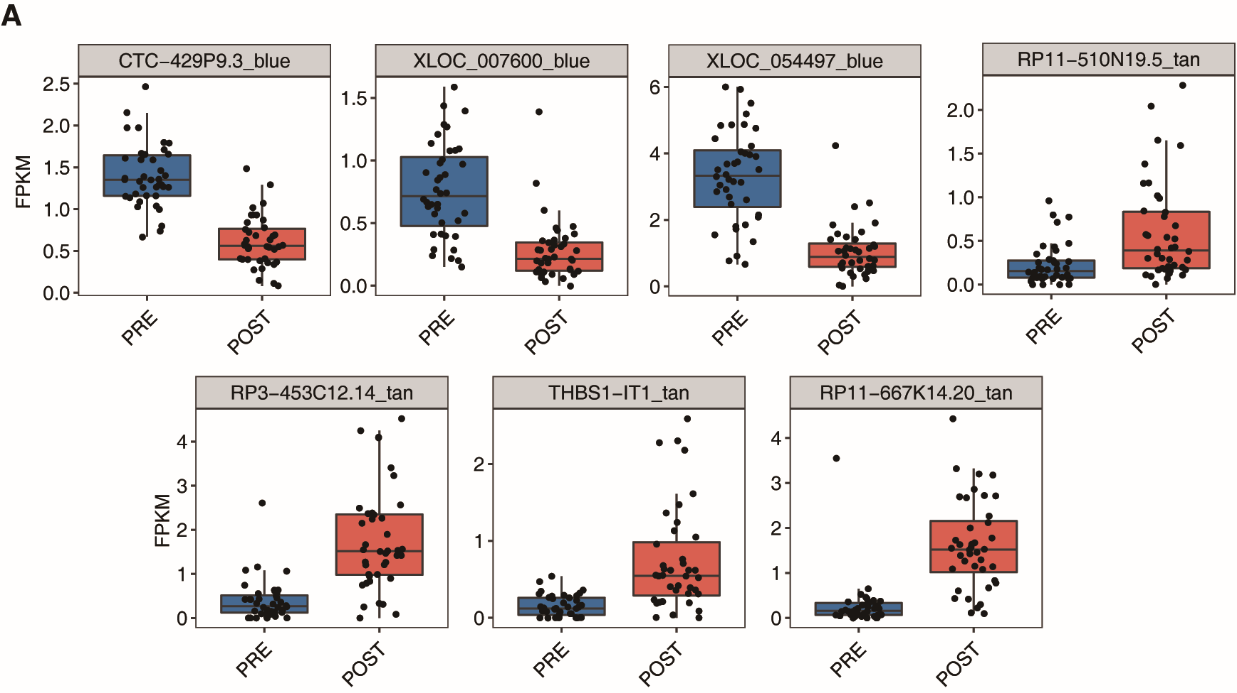


**Figure S4. Genes involved in apoptosis pathway were largely up regulated by 12 immune associated DElncRNAs after liver transplantation.**

A. Box plot showing expression profile of 7 immune-related lncRNAs.
